# Thermal Characteristics and Bacterial Diversity of Forest Soil in the Haean Basin of Korea

**DOI:** 10.1155/2014/247401

**Published:** 2014-11-10

**Authors:** Heejung Kim, Jin-Yong Lee, Kang-Kun Lee

**Affiliations:** ^1^School of Earth and Environmental Sciences, Seoul National University, Seoul 151-747, Republic of Korea; ^2^Department of Geology, Kangwon National University, Chuncheon 200-701, Republic of Korea

## Abstract

To predict biotic responses to disturbances in forest environments, it is important to examine both the thermophysical properties of forest soils and the diversity of microorganisms that these soils contain. To predict the effects of climate change on forests, in particular, it is essential to understand the interactions between the soil surface, the air, and the biological diversity in the soil. In this study, the temperature and thermal properties of forest soil at three depths at a site in the Haean basin of Korea were measured over a period of four months. Metagenomic analyses were also carried out to ascertain the diversity of microorganisms inhabiting the soil. The thermal diffusivity of the soil at the study site was 5.9 × 10^−8^ m^2^ *·*s^−1^. The heat flow through the soil resulted from the cooling and heating processes acting on the surface layers of the soils. The heat productivity in the soil varied through time. The phylum Proteobacteria predominated at all three soil depths, with members of Proteobacteria forming a substantial fraction (25.64 to 39.29%). The diversity and richness of microorganisms in the soil were both highest at the deepest depth, 90 cm, where the soil temperature fluctuation was the minimum.

## 1. Introduction

The health of forests has been a key concern in recent years because of natural disasters caused by environmental pollution [1–4], climate change [5–8], and ecological destruction [9–11]. Furthermore, deforestation and the degradation of natural forest environments have been central topics in United Nations venues such as the Framework Convention on Climate Change (UNFCCC), the Convention on Biological Diversity (UNCBD), and the Convention to Combat Desertification (UNCCD), marking the integrity of forest ecosystems as one of the most pressing ecological issues worldwide.

Forests provide a wide variety of important ecosystem services, including headwater conservation [12], water purification [13], soil erosion prevention [14, 15], landslide prevention [16, 17], carbon dioxide absorption [18], atmospheric purification [19, 20], conservation of native animal species diversity [21], and the protection of woodland animals [22].

Forest area of the Republic of Korea (hereafter Korea) was 6,369,000 ha in 2010, accounting for 63.7% of its total land area—the fourth-highest proportion among OECD countries. However, its forest area has been shrinking, falling from 6,640,839 ha in 1974 to 6,394,000 ha in 2005, 6,382,000 ha in 2007, and 6,370,000 ha in 2009 [23]. This decrease has been caused mainly by increasing demand for land, itself a result of increasing population and numerous development projects [24]. Even as Korea's forest area continues to decrease, however, the forest area of other advanced countries is increasing. In Germany, a model country for forest preservation, the forest area has increased from 10,740,000 ha to 11,076,000 ha [25]. In the United States, it has increased from 225,993,000 ha to 303,089,000 ha, an increase of 77,096,000 ha [25, 26].

In this study, we investigated the thermal characteristics of forest soil at a site in the Haean basin of Korea. Forests are susceptible to climate change, and the evidence for global climate change has been reported in many studies [27–31]. We also analyzed the heat transfer characteristics of the soil, using time-series temperature data from the winter when the surface layer of soil in the forest freezes. Finally, we assessed the microbial diversity in the soil, at three sampling depths, using metagenomic analyses.

Heat transfer in forest soil is very complex and depends on factors such as conduction, heat production caused by phase transitions undergone by the water in the soil, and transfer of water and vapor between the soil and the atmosphere. Heat conduction is a key mechanism causing heat transfer within soil and is particularly important during the winter, although nonconductive heat transfer driven by the convection of groundwater can also occur if there is a suitable temperature gradient in the groundwater. The thermal diffusivity of the soil at the study site was 5.9 × 10^−8^ m^2^·s^−1^. The heat flow through the soil resulted from the cooling and heating processes acting on the surface layers of the soils. The phylum Proteobacteria dominated at all three soil depths, with members of Proteobacteria forming a substantial fraction (25.64 to 39.29%) of the 16S rRNA gene sequences in all samples. In this study, in order to determine the thermal characteristics of forest soil, we used soil temperature measurements to investigate the heat transfer process by analyzing the temperature, thermal diffusivity, and heat production rate of the soil.

## 2. Methods and Materials

### 2.1. Study Site

**Figure 1:**
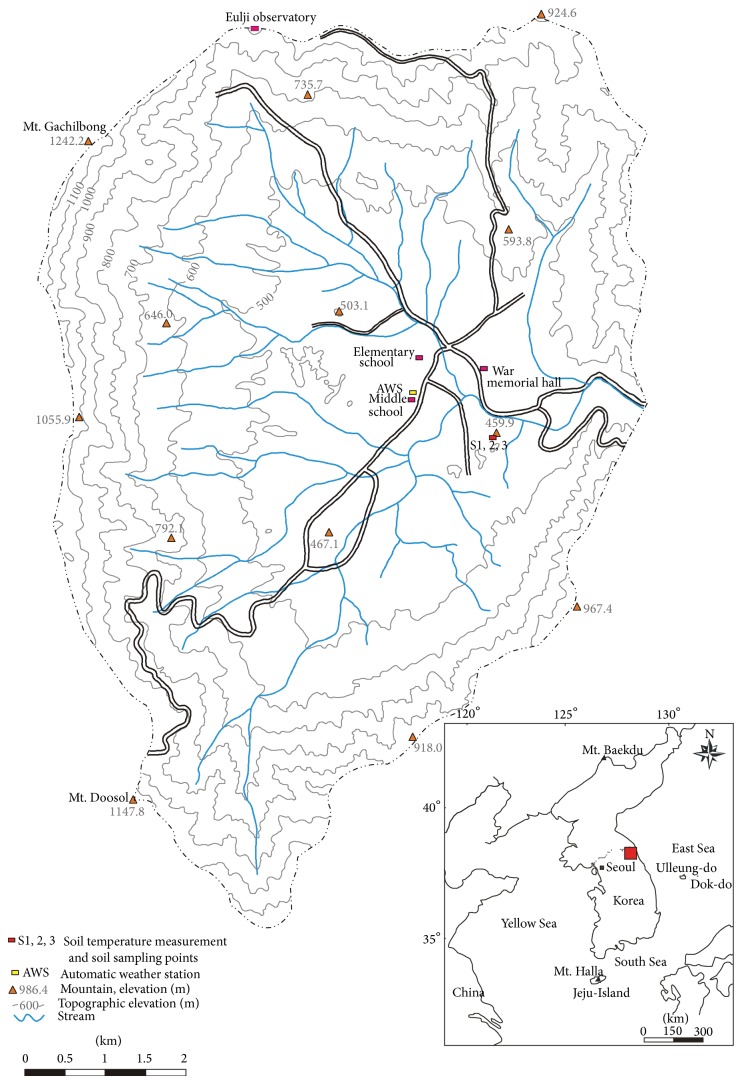
Location of the study site.

The Haean basin is located in the central region of the Korean peninsula, spanning latitudes of 38°15′–38°20′N and longitudes of 128°15′–128°10′E (Figure 1). It is 19 km northwest of Inje-gun, Gangwon-do, and 26 km northeast of Yanggu-eup, Yanggu-gun, and is part of the Haean-myeon, Yanggu-gun, and Gangwon-do administrative districts, which border the basin. This is a rugged, mountainous region, but the Haean basin itself is a relatively flat, oval-shaped area.

**Figure 2:**
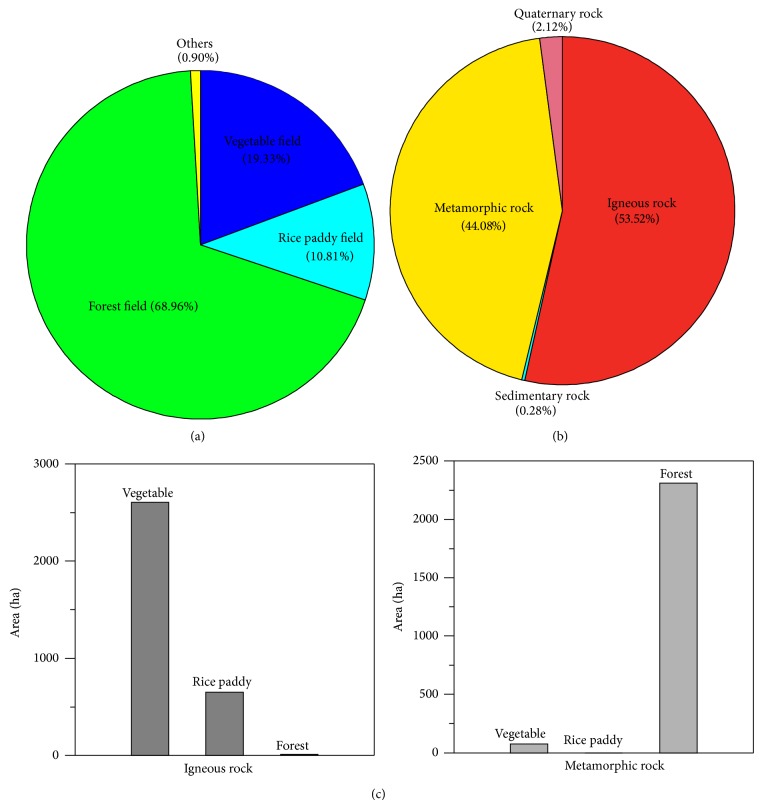
Land uses and soil parent materials in the Haean basin, Korea.

The basin is located in the northeast of the Gyeonggi metamorphic rock complex, a region composed of Precambrian metamorphic rock (gneiss, mica-schist, and quartzite) and Jurassic igneous rock (granite) that is interpenetrated with metamorphic rock [32, 33] (Figure 2). The basin was formed by differential erosion [32, 34]; the outskirts of the basin are predominantly the harder metamorphic rocks, whereas the interior of the basin is predominantly granite, which is more vulnerable to weathering. Much of the granite is thus exposed as saprolite, which gradually turns into granodiorite and diorite [32, 35], and the soil in the basin is derived from these rocks. The basin varies in altitude from 400 m to 1,304 m, with an average altitude difference of 400–500 m between the basin bed and surrounding ridge [36].

The average slope for the basin area as a whole is 11°, but the steepest slopes are generally in the vicinity of the surrounding ridge, with an average slope of 20°, whereas the basin bed has an average slope of only 5°. The slopes of the basin decline monotonically from ridge to bed, and thus, the basin is bowl-shaped and concave. Land use in the basin is varied; the higher altitudes, with steeper slopes, are forested, whereas the flatter areas at middle and low altitudes consist of fields and rice paddies (Figure 2). Overall, 70% of the Haean basin consists of forest. However, farming area in the basin is increasing, and so the forest area is decreasing [33, 36]. From 2002 to 2011, the maximum and minimum of annual mean air temperatures of the basin were 25.3°C and −11.5°C, respectively, and their mean was 10.1°C [36].

### 2.2. Soil Temperature, Thermal Diffusivity, Heat Flow, and Heat Production

Soil temperature was measured every 30 minutes using an iButton (Maxim, model DS1921G) that was installed at depths of 30, 60, and 90 cm in the Haean basin forest soil. This device is able to measure temperatures from −40°C to 80°C with a resolution of 0.01°C. Measurements were taken without interruption from November 11, 2011, to February 14, 2012, which is winter season in Korea. The temperature ranges from −1.6 to 8.4°C at 30 cm depth, −1.1 to 7.9°C at 60 cm depth, and 0.8 to 9.6°C at 90 cm depth, respectively.

From these measurements, other thermophysical properties of the soil were derived using standard methods summarized here. The law of conservation of energy can be expressed as

(1) $$\begin{array}{c} \frac{\partial }{\partial t}\left[cT\right]+\nabla \cdot \vec{q}=A, \end{array}$$

where *c* = heat capacity, *T* = temperature (°C), $\vec{q}$ = heat flux, and *A* = heat production rate.

Heat flow, according to Fourier's law, is expressed by

(2) $$\begin{array}{c} \vec{q}=-k\nabla T. \end{array}$$

The heat transfer equation can then be derived from (1) and (2) to yield

(3) $$\begin{array}{c} \nabla^{2}T+\frac{A}{k}=\frac{1}{\alpha }\frac{\partial T}{\partial t}, \end{array}$$

where *k* = thermal conductivity and *α* = thermal diffusivity.

When (3) is solved, with an initial temperature of 0°C, for the surface temperature *T* over the time interval (0, *t*) in a semi-infinite medium in which the production and extinction of soil heat do not occur, its solution has been found to be the convolution of the surface temperature function and the heat transfer function (*f*_*τ*_) [37]:

(4) $$\begin{array}{c} T\left(z,t\right)=\overset{t}{\underset{0}{\int }}T\left(0,\tau \right)f_{T}\left(t-\tau ,z\right)d\tau . \end{array}$$

Here, the heat transfer function is

(5) $$\begin{array}{c} f_{T}\left(t,z\right)=\frac{z}{2{\left[\pi \alpha t^{3}\right]}^{1/2}}\exp\left(-\frac{z^{2}}{4\alpha t}\right). \end{array}$$

Accordingly, the change in temperature of subsurface soil, which occurs only by heat conduction in a uniform medium without production or extinction of soil heat, can be calculated using (4) and (5). In addition, since we performed soil temperature measurements at discrete depths, a finite-difference method approximation to (2) can be used to calculate heat flow by depth as

(6) $$\begin{array}{c} q=-k\frac{T\left(z_{i+1},t\right)-T\left(z_{i},t\right)}{z_{i+1}-z_{i}}. \end{array}$$

Here, the heat production rate at [*z*_*i*_, *z*_*i*+1_]×[*t* ^*j*^, *t* ^*j*+1^], a unit section in depth and time, can be obtained using Roth and Boike's [38] equation [39]. Consider

(7) A=1tj+1−tjzi+1−zi×∫tjtj+1∫zizi+1Az,tdzdt=1tj+1−tjzi+1−zi×c∫zizi+1Tz,tj+1−Tz,tjdz  −α∫tjtj+1T′zi+1,t−T′zi,tdt,

where *T*′ = differential difference according to the depth of soil heat.

### 2.3. Metagenomic Analysis

Soil samples were collected for microbial analysis from three depths (30, 60, and 90 cm) from the study site in December, 2012. DNA was extracted from the samples using the FastDNA SPIN Kit for Soil (MP Biomedicals, #116560-200). 16S rRNA genes were amplified by ChunLab (Seoul, Korea) using PCR with forward and reverse primers prior to sequencing [40, 41]. The amplification conditions for PCR were (i) an initial denaturation step at 94°C for 5 min, (ii) 30 cycles of denaturation at 94°C for 30 sec followed by annealing at 55°C for 45 sec, and (iii) an extension step at 72°C for 90 sec.

Pyrosequencing of the amplified 16S rRNA was then conducted using a 454 GS FLX Titanium Junior (Roche, NJ, USA) by ChunLab (Seoul, Korea). Distinct sequences were deposited in the Sequence Read Archive (NCBI) by ChunLab. Bacterial community structures were analyzed using operational taxonomic units (OTUs). Distance matrices were used to define OTUs for calculation of the abundance-based coverage estimator (ACE), the Chao 1 richness estimator [42], Shannon and Simpson diversity indices, and rarefaction curves. Each sequence was identified by comparing it with sequences in the EzTaxon-extended database (ChunLab, eztaxon-e.org) using BLASTN searches and pairwise similarity comparisons [43].

### 2.4. Phylogenetic Analysis

For phylogenetic analysis of the dominant bacterial communities present in the forest soil samples, we used the 15 OTUs that were present at an abundance of at least 1%, out of the 274 OTUs (5,544 sequences) that appeared at all three sampling depths. Additionally, we used 34 OTUs that were present at an abundance of at least 0.5% in the individual samples from depths of 30 cm (10 OTUs), 60 cm (15 OTUs), and 90 cm (9 OTUs). Sequences of these 49 dominant OTUs and their related neighbors were downloaded, together with those of* Desulfurococcus kamchatkensis* 1221n (NR_074374),* Acidilobus aceticus* 1904 (NR_041774), and* Halobaculum gomorrense* (L37444), which were used as outgroups.

We aligned the 49 dominant sequences with reference sequences using BioEdit version 7.0.9.0 [44]. Phylogenetic trees were constructed from the aligned sequences using three methods: (1) neighbor-joining (NJ [45]) using the Kimura 2-parameter distance model, (2) maximum likelihood (ML) using the Tamura-Nei distance model, and (3) Bayesian inference (BI) by the Markov Chain Monte Carlo (MCMC) method using the software package Molecular Evolutionary Genetics Analysis (MEGA) version 5.10 [46] and MrBayes version 3.1.2 [47]. The stability of branches was assessed using a bootstrap analysis with 1,000 replicates.

## 3. Results and Discussion

### 3.1. Soil Temperature and Thermal Diffusivity

**Figure 3:**
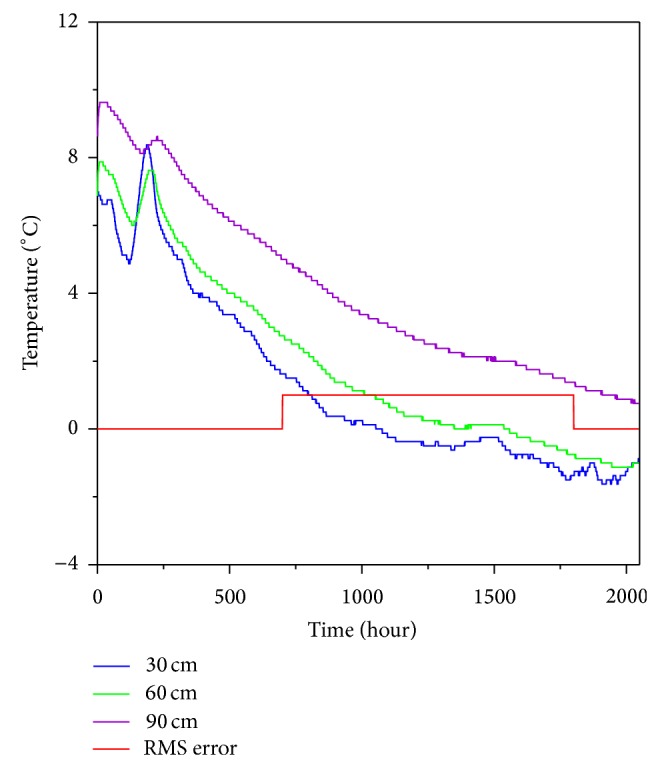
Soil temperatures at the study site for depths of 30, 60, and 90 cm from November 11, 2011, to February 14, 2012.

The mean soil temperatures, with associated standard deviations, at the three soil depths measured are shown in Table 1, while changes in the soil temperature through time are shown in Figure 3. In general, shallower soil depths are expected to show greater temperature variation. At our study site, however, the standard deviation of the temperate measurements was greatest at a depth of 60 cm. The temperature variation of shallow depth soils is generally determined by the diurnal air temperature fluctuations, wind speed, and the soil constituents, which include organic matter, mineral, water, and air. In comparison with soil with much organic matter, mineral soil tends to have higher thermal conductivity, thermal admittance, and thermal diffusivity [48, 49]. In this site, more organic matters were found at 30 cm depth than at 60 cm depth. As a consequence, a mineral soil at 60 cm depth tends to undergo large fluctuations of temperature than 30 cm depth.

The volumetric soil heat capacity can be determined by sum of the specific heats of the soil constituents [50]. The differences of soil constituents make the variation of the soil temperature through the depths. Furthermore, the shallower soils are covered to vary with vegetation. The above assessment of soil temperature variation, while not intended to be comprehensive, suggests that temperature of shallower soil might be affected by the above factors more than 60 cm depth. The increase in temperature variation in 30 cm depth was regarded as a primary result of the organic dry soil rapidly cooling and heating by air temperature fluctuations and wind speed. Moreover, the vegetations might be able to extract moisture from 30 cm depth so it can show a temperature variation.

Changes in soil temperature can essentially be divided into four periods of time: the isothermal, cold, warming, and thawing periods [38]. In the isothermal period, the soil temperature is almost constant because the soil is in the process of freezing. In the cold period, the soil maintains a very low temperature after having completely frozen. In the warming period, the soil temperature increases prior to the initiation of melting. Finally, the thawing period spans from the initiation of melting, which starts at the ground surface, to the completion of melting. The data in this study were collected during the isothermal period and the subsequent cold period. Accordingly, they show a decreasing soil temperature trend that is accounted for by seasonality at the study site.

**Figure 4:**
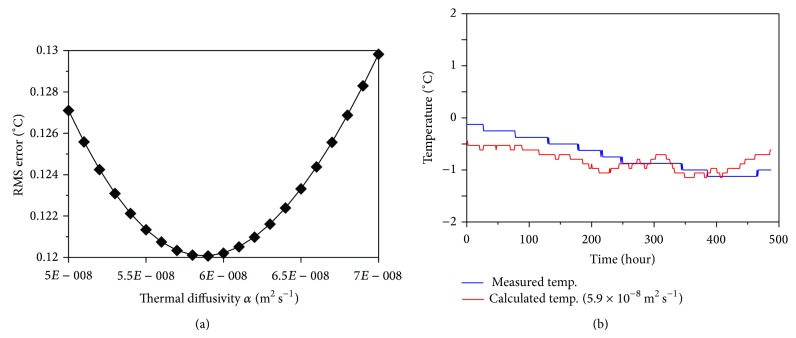
RMS errors between the measured and predicted temperatures to the thermal diffusivity values for (a) a soil depth of 60 cm. Panel (b) shows best fits to this data. The RMS errors were calculated using measurements taken during the period when conduction was the dominant mode of heat transfer.

Since soil temperatures in the cold period are below freezing by definition, phase transitions of soil water are unimportant, resulting in a heat transfer process that mainly depends on heat conduction [51]. Therefore, subsurface temperatures can be estimated from the ground surface temperature using (4) and (5) and the thermal diffusivity can then be determined, allowing the differences between estimated and actual temperatures to be minimized. There was no existing ground surface temperature data for our study site; therefore, instead we estimated the soil temperature at 60 cm depth using the measured soil temperature at 30 cm depth and then calculated the RMS error between the estimated and measured 60 cm depth temperatures to determine the thermal diffusivity (Figure 4). This calculation was based on temperature measurements taken during the cold period, when the soil temperature was below 0°C (marked by the red line in Figure 3). The RMS error analysis revealed that the optimal thermal diffusivity was 5.9 × 10^−8 ^m^2^·s^−1^ at a depth of 60 cm. The thermal diffusivity of a previous study was 4.0 × 10^−7 ^m^2^·s^−1^ in Mt. Jumbong forest (10 cm depth soil [52]), near our study site. This value is greater than ours, which indicates the thermal diffusivity of forest soil is affected by not only soil constituents but also soil depth.

### 3.2. Heat Flow and Latent Heat

Heat flow at the ground surface can be calculated using a finite-difference method approximation as in (6). The heat flow through time, obtained by applying the previously determined thermal diffusivity of 5.9 × 10^−8 ^m^2^·s^−1^ in this equation, is depicted in Figure 4 with the soil's average heat flow of 2.2 × 10^6^ J·m^−3^·K^−1^. In general, negative heat flow means that the local soil temperature is decreasing, while positive heat flow means it is increasing [53]. While heat transfer by heat conduction plays an important role in the process of transferring heat into the subsurface, in forest soil heat transfer by the phase change and convection of pore water also plays a central role. The production and extinction of latent heat occur during phase changes between ice, water, and vapor. In addition, water and vapor can move through the soil by convection and diffusion and can then cool and refreeze, thereby discharging latent heat in a new location [54]. Therefore, the effects of this process of phase change, convection, and diffusivity can be quantified as a heat production rate, which can be estimated using (7).

**Figure 5:**
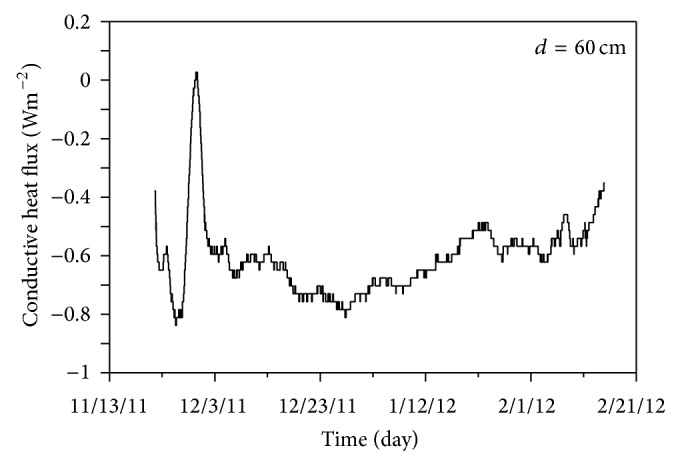
Heat flux estimated by the finite-difference approximation for 60 cm depth.

The integral in (7) can be calculated by numerical differentiation by the depth of temperature and then numerical integration by the time and depth. Since numerical differentiation uses centered finite differences, soil temperature data at four depths [*z*_*i*−1_, *z*_*i*_, *z*_*i*+1_, *z*_*i*+2_] is needed in order to calculate the heat production rate at a single section [*z*_*i*_, *z*_*i*+1_]. We measured temperatures at depths of 30, 60, and 90 cm, and therefore the heat production rate at a depth of 60 cm could be calculated. The calculated change in heat production rate over time is shown in Figure 5. Since the duration of the monitoring period was relatively short, heat flow within the soil appeared essentially constant. Accordingly, the changes in the heat flow we observed are presumably due to seasonal climatic variation and the local cooling and heating processes at the ground surface.

### 3.3. Bacteria Richness and Diversity Indices

**Figure 6:**
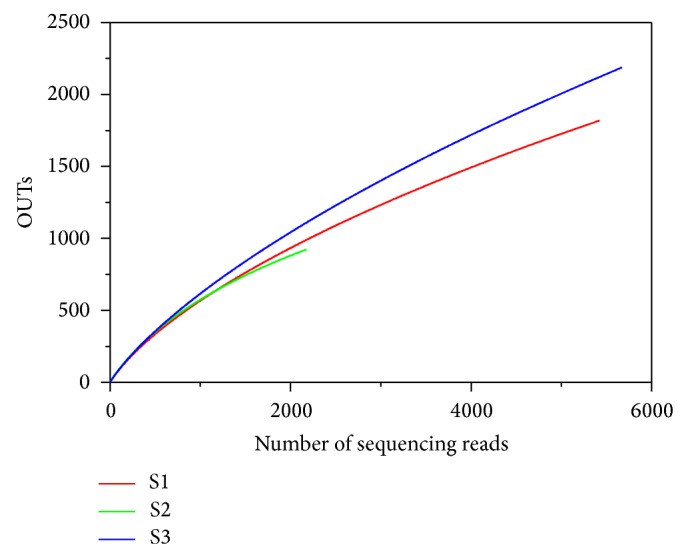
Rarefaction curves indicating the observed number of OTUs within the 16S rRNA gene sequences at the three depths sampled (S1 = 30 cm, S2 = 60 cm, and S3 = 90 cm).

To determine rarefaction curves, richness, and diversity, we identified operational taxonomic units (OTUs) in each sequencing read. The rarefaction analysis of bacterial communities derived from the three different sampling depths (S1, S2, and S3) is depicted in Figure 6. In addition, a comparison of the rarefaction analysis with the number of OTUs estimated by the Chao 1 richness estimator revealed that 73.6 to 78.6% of the estimated taxonomic richness was covered by the sequencing effort (Table 2). The Shannon index of diversity (*H*′) was also determined for all samples (Table 2) and ranged from 6.40 to 6.82 among the different depths. Bacterial diversity is generally expected to decrease with increasing soil depth. Here, however, the Shannon diversity index *H*′ was highest at the deepest depth (S3). The shallower soil tends to undergo larger and more rapid temperature responses to surface temperature fluctuations. The temperature fluctuation is often an important limitation of the soil biological activity. Ballard [55] found that changes in temperature are likely to be significant in biological activity and its little change may enhance the biological diversity and richness. The higher bacterial diversity at that depth (90 cm) is consistent with the least temperature variation and thus it can be inferred that the temperature is one of the key factors to determine the bacterial diversity and richness [56].

### 3.4. Distribution of Taxa and Phylotypes across Samples

**Figure 7:**
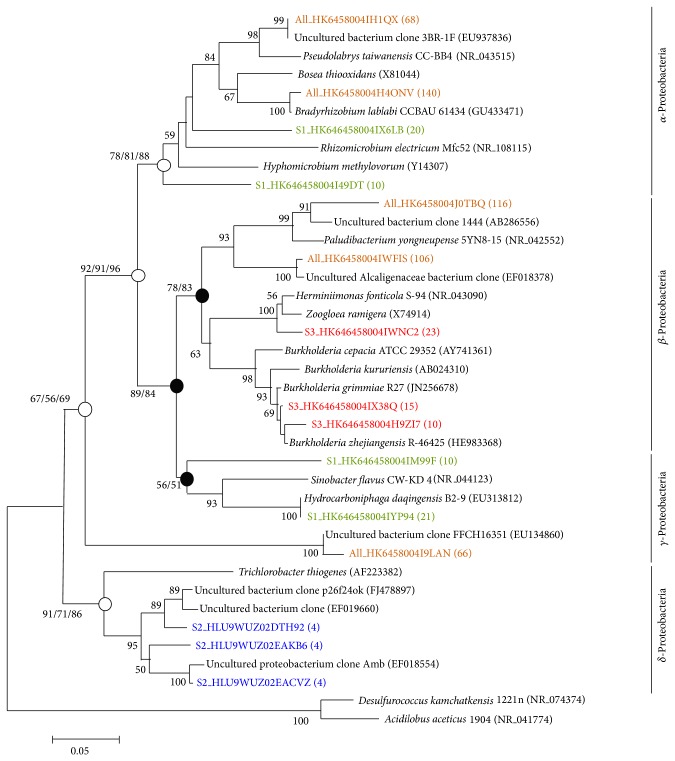
Neighbor-joining phylogenetic tree showing the dominant bacterial relationships associated with phylum Proteobacteria in the soil samples. The bar in the bottom represents 0.05 nucleotide substitutions per nucleotide position.

**Figure 8:**
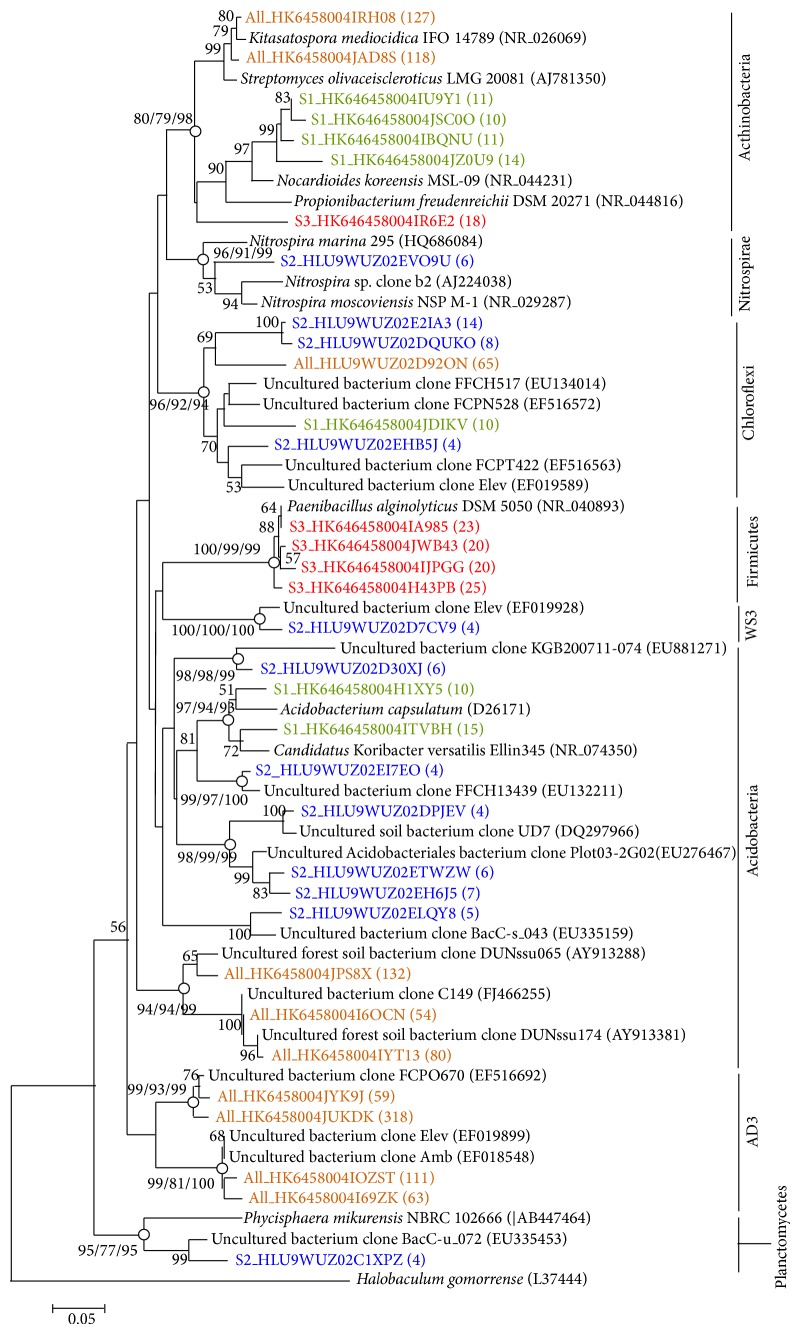
Neighbor-joining phylogenetic tree showing the dominant bacterial relationships associated with phyla other than Proteobacteria in the soil samples. The bar in the bottom represents 0.05 nucleotide substitutions per nucleotide position.

Figure 7 shows a phylogenetic tree generated by the neighbor-joining method that depicts the dominant bacterial relationships from the forest soil associated with the phylum Proteobacteria. Bootstrap values (>50%) based on 1,000 replicates are shown. An open circle indicates that the corresponding branch was recovered with a high bootstrap value by all three tree generation methods (neighbor-joining, maximum likelihood, and Bayesian inference), whereas a closed circle indicates that the corresponding branch was recovered only by the neighbor-joining and maximum likelihood methods.* Desulfurococcus kamchatkensis* 1221n (NR_074374) and* Acidilobus aceticus* 1904 (NR_041774) were used as the outgroups. Colors represent the dominant bacterial sequences obtained from the soil samples at various depths: 30 to 90 cm, orange; <30 cm, green; <60 cm, blue; and <90 cm, red.  Figure 8 shows a phylogenetic tree generated by the neighbor-joining method that depicts the dominant bacterial relationships from the forest soil associated with phyla other than Proteobacteria.* Halobaculum gomorrense* (L37444) was used as an outgroup.

The 5,544 sequences were affiliated with 40 phyla across the entire data set. The dominant phyla within S1 were Proteobacteria, Acidobacteria, Actinobacteria, Chloroflexi, Gemmatimonadetes, Planctomycetes, Nitrospirea, Cyanobacteria, Firmicutes, and Bacteroidetes, representing 39.29, 27.03, 7.65, 3.94, 2.04, 1.55, 1.27, 1.18, 1.05, and 0.96% of the sequences, respectively. The dominant phyla within S2 were Acidobacteria, Proteobacteria, Chloroflexi, Actinobacteria, Gemmatimonadetes, Nitrospirea, Verrucomicrobia, Planctomycetes, Thermobaculum_p, and Bacteroidetes, representing 32.55, 25.64, 7.37, 6.63, 4.56, 3.31, 2.39, 1.70, 1.34, and 1.01% of the sequences, respectively. The dominant phyla across S3 were Proteobacteria, Acidobacteria, Actinobacteria, Firmicutes, Chloroflexi, Gemmatimonadetes, Nitrospirea, Verrucomicrobia, Cyanobacteria, Planctomycetes, and Bacteroidetes, representing 31.91, 21.21, 14.35, 4.86, 3.23, 2.13, 1.74, 1.55, 1.32, 1.28, and 1.12% of the sequences, respectively.

The phylum Proteobacteria was thus predominant across the S1, S2, and S3 samples taken together, and its members comprised a substantial percentage (25.64 to 39.29%) of the 16S rRNA gene sequences at all of the depths sampled. The phylum Proteobacteria is divided into 4 subgroups (*α*, *β*, *γ*, and *δ*-Proteobacteria). Members of the phylum Acidobacteria were also very common in all samples, comprising a substantial percentage (21.21 to 32.55%) of the 16S rRNA gene sequences at all of the depths sampled. This abundance of Acidobacteria is in accord with other studies [57] of the composition of soil-derived bacterial communities from a variety of environments, such as forests, grasslands, and agricultural areas. Although we covered 5,544 sequences, we did not examine the full extent of bacteria richness at the phylum level within various depth soils. In this case, the S2 soil showed a lower estimated bacterial diversity than the S1 and S3 soils. Thus, it can be inferred that the S1 soil is more plentiful of organic matter than S2 and S3 soil is more stable than S1 and S2 soils with respect to temperature variation.

## 4. Conclusions

Thermal characteristics and microbial diversity were examined in the forest soil of the Haean basin of Korea. The thermal diffusivity of the soil was 5.9 × 10^−8 ^m^2^·s^−1^ at a depth of 60 cm. Heat flow was negative at depths where the soil temperature was decreasing, whereas heat increased when the internal temperature of the soil was higher than the ground surface temperature. Analysis of microbial diversity revealed that diversity and species richness varied with depth. In particular, the microbial species richness was highest at the deepest depth sampled, 90 cm, a result that is contrary to the usual relationship between microbial species richness and soil depth. The reason for this appeared to be that the soil at 90 cm depth maintained a relatively steady temperature, allowing a high species richness and diversity, compared to the shallower depths (30~60 cm) where the temperature fluctuated sharply. Studies of the thermophysical properties and microbial diversity of forest soils are valuable since they can help predict the biological diversity of forest ecosystems, allowing us to evaluate the effects of climate change on these ecosystems in the coming years.

## Figures and Tables

**Table 1:** Summary statistics for temperature measurements from the study site. S1, S2, and S3 indicate the three soil depths at which measurements were taken.

Point	Depth (cm)	Mean temperature (°C)	Standard deviation (°C)
S1	30	1.30	2.63
S2	60	1.99	2.65
S3	90	4.12	2.59

**Table 2:** Species richness estimates obtained from the soil samples.

Sample	Rarefaction (number of OTUs)	Shannon index (*H*′)	Chao 1 (number of OTUs)	Coverage (%)
S1	1818	6.59	4331	78.6
S2	922	6.40	1625	76.4
S3	2189	6.82	6032	73.6
